# Cranial Shape Measurements Obtained Using a Caliper and Elastic Bands Are Useful for Brachycephaly and Deformational Plagiocephaly Screening

**DOI:** 10.3390/jcm12082787

**Published:** 2023-04-09

**Authors:** Taishin Maedomari, Hiroshi Miyabayashi, Yukari Tanaka, Chihiro Mukai, Aya Nakanomori, Katsuya Saito, Risa Kato, Takanori Noto, Nobuhiko Nagano, Ichiro Morioka

**Affiliations:** 1 Department of Pediatrics and Child Health, Nihon University School of Medicine, Tokyo 173-8610, Japan; 2 Department of Pediatrics, Kasukabe Medical Center, Saitama 344-8588, Japan

**Keywords:** cranial shape, three-dimensional scanner, brachycephaly, deformational plagiocephaly, infant

## Abstract

We assessed a method for screening the cranial shape of 1-month-old infants using a simple measuring instrument instead of a three-dimensional scanner. The Mimos craniometer was used to measure cranial length, cranial width, and two diagonal lengths to calculate the cranial index (CI) and cranial asymmetry (CA). We defined a CI > 90% as brachycephaly and CA > 5 mm as deformational plagiocephaly (DP). Intra- and inter-examiner accuracy analyses were performed on a dummy doll and 1-month-old infants. The measurements of healthy 1-month-old infants were compared with previously reported three-dimensional scanner measurements. Intra- and inter-rater measurements showed good accuracy; diagnostic accuracy comparisons of brachycephaly and DP using a three-dimensional scanner showed kappa values of 1.0 and 0.8, respectively. Comparisons were made among 113 infants matched for day-age at the date of measurement; there were no significant differences in the CI (85.0% vs. 85.2%, *p* = 0.98) and CA (5.9 mm vs. 6.0 mm, *p* = 0.48) between the scanner and caliper measurements, nor in the prevalence of brachycephaly (12.4% vs. 17.7%, *p* = 0.35) or DP (58.4% vs. 56.6%, *p* = 0.89). This simple measurement method using calipers and bands was useful in screening for brachycephaly and DP in 1-month-old infants.

## 1. Introduction

The human skull expands significantly during infancy. Infant skulls are soft and flexible because of rapid brain development, particularly during the first few months of life when the sutures are open and head growth occurs the fastest. Infants are exposed to external influences during this crucial time of cranial development, including gravity, orientation, and sleeping position, which passively alter the cranial shape. The incidence of deformational plagiocephaly (DP) has increased considerably because of the American Academy of Pediatrics’ recommendation that infants should be placed in a supine position (Back to Sleep campaign) [1,2]. DP is not only a cosmetic issue but has also been reported to have negative developmental effects [3,4,5]. Therefore, measurement and diagnostic tools for cranial shapes in infants are essential.

The prevalence of DP in Canada is reported to be 46.6% [6]. Our previous report showed that the prevalence of DP among Japanese infants at 3 months of age was 56.5% [7]. Japanese infants reportedly have a higher prevalence of DP than infants in other countries, and approximately 5% of healthy infants require helmet therapy for DP in Japan [7,8]. Additionally, the prevalence of brachycephaly (BC) is higher in Japan than in other countries, and diagnostic criteria specific to Japanese infants are necessary [9,10].

Currently, a handheld three-dimensional (3D) scanner (3DS) is used in Japan to detect cranial shapes. However, medical institutions have not widely adopted these devices owing to their high cost. A limited number of medical facilities can diagnose abnormal cranial shapes and perform helmet therapy in infants who potentially require interventions (5% of healthy infants) [7,10]. One reason for this may be a lack of awareness of the importance of cranial geometry. In Japan, the negative effects of DP are not well known. Many infants are not currently examined for abnormal cranial shapes during the first month of life; this is a crucial period because the cranial shape at one month is a good predictor of DP severity at 6 months [7].

Herein, we investigated the possibility of measuring cranial shape and screening BC and DP during the first month of life using simple elastic bands and a caliper instead of a 3DS.

## 2. Materials and Methods

### 2.1. Participants

Infants who underwent an examination at one month of age at the Kasukabe Medical Center and Nihon University Itabashi Hospital between 5 April 2022, and 5 February 2023, were included in the study. The exclusion criteria included non-Japanese races, preterm infants, those with neonatal asphyxia, surface malformation, and those with congenital diseases. Plagiocephaly was not an exclusion criterion. No infant was diagnosed with DP before the 1-month checkup. This study was approved by the Ethics Committee of Kasukabe Medical Center (2022-001; approval date: 4 April 2022; c2022-043; approval date, 5 January 2023). Written consent was obtained from the parents of the infants.

The comparison control group for the current study was infants who had cranial shape measured with 3DS at 1 month of age based on our database of previous reports [7,11]. These controls were infants who visited our institution between April 2020 and April 2021 at 1 month of age and underwent 3D measurement, and the exclusion criteria were in accordance with the exclusion criteria of the present study.

### 2.2. Methods

#### 2.2.1. Parameter Calculation Using the Caliper Method

**Figure 1:**
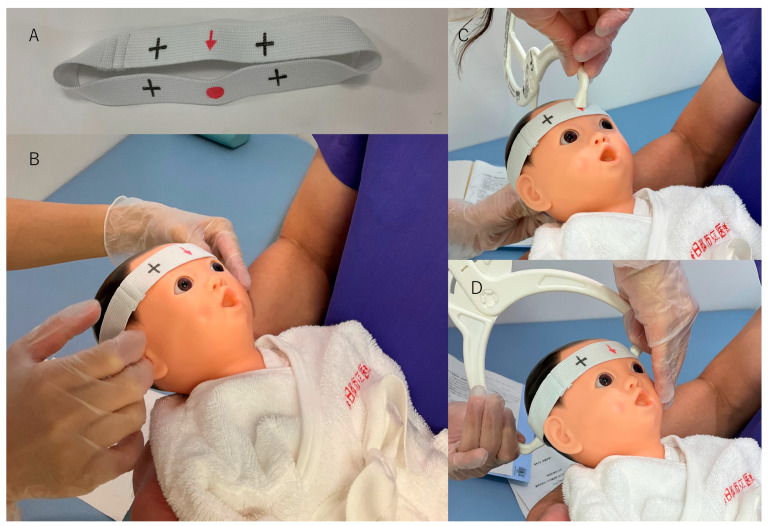
Procedure for measurement using calipers and elastic bands. (**A**) The elastic band included in the Mimos craniometer. (**B**) Elastic bands are placed with the “↓” symbol between the eyebrows and the “●” symbol at the protuberantia occipitalis externa. (**C**) Cranial length is the length between “↓” and “●” on the band. (**D**) The length of the longer diagonal of the “+” symbols measured diagonally is designated as diagonal A.

Cranial shape measurements were performed using a Mimos craniometer (Think Pipe Line SLU, Manresa, Spain), caliper, and elastic bands. An elastic band with a circumference of 310 mm and with “↓” and “●” symbols at both ends was used. It has four “+” symbols approximately 25 mm from each side of the “↓” and “●” symbols (Figure 1A). This elastic band was placed carefully with the “↓” symbol between the eyebrows and the “●” symbol at the protuberantia occipitalis externa (Figure 1B), and the measurements were recorded. The cranial length (CrL) was the length between “↓” and “●” on the band (Figure 1C), and the cranial width (CrW) was the maximum width of the band measured using the caliper.

The length of the longer diagonal of the “+” symbols (Figure 1D) was designated as diagonal A (LDA). Similarly, the shorter diagonal was designated as diagonal B (SDB). The cephalic index (CI) was calculated as CrW/CrL × 100 [2]. Cranial asymmetry (CA) was calculated as LDA minus SDB [2]. In this study, the CI and CA, which are easy to compute, were used for screening purposes. The diagnostic threshold for BC in Japanese was reported to be a CI > 93.2% [9], and DP was defined as having a cranial vault asymmetry index > 5% [7,8]. However, in this study, for ease of discrimination, the diagnostic thresholds for BC and DP were determined to be >90% and >5 mm, respectively [8,12].

#### 2.2.2. Parameter Calculation from 3D Images

**Figure 2:**
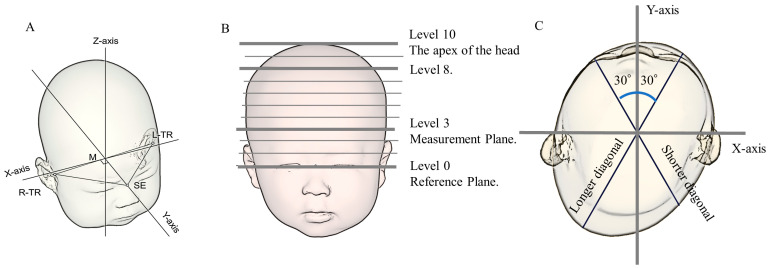
The measurement of each parameter using the 3D scanner. This figure is a modification of the figure created in our previous study [7]. (**A**) Determination of the reference plane (level 0) and *X*-, *Y*-, and *Z*-axes. (**B**) Determination of the measurement plane. (**C**) Cross-sectional view of level 3 of the measurement plane. The *X*-axis, *Y*-axis, and 30° diagonals were used to quantify cranial asymmetry and cranial vault asymmetry index. L-TR, left tragion; M, midpoint of both tragions; R-TR, right tragion; SE, sellion; TR, tragion.

The 3D standard triangulated language (STL) data created by 3DS were analyzed using Artec Studio image analysis software (Artec, Inc., Luxembourg, Luxembourg) and original analysis software from Japan Medical Company, Inc. (Tokyo, Japan). Figure 2 shows the method used to determine the parameters [7].

The plane connecting the three points of the sellion (the lowest point of the nasal root) and the left and right tragions (the upper margins of the tragus) was used as the reference plane (level 0). The software identified the midpoints of the left and right tragion. A line passing through the two points of the sellion and midpoint was defined as the *Y*-axis; the *X*-axis was a line perpendicular to the *Y*-axis at the midpoint on the level 0 plane (Figure 2A) [13,14]. The software constructed 10 equal cross-sections on the cranium from level 0. The height of each cross-section was determined by categorizing the height from the level 0 plane to the apex of the head into 10 equal parts (Figure 2B). The level 3 plane was used as the measurement plane [14,15]. Figure 2C shows the cross-sectional view of the measurement plane. CrL and CrW were measured as the lengths of the planes in the *Y*-axis and *X*-axis directions, respectively. Additionally, diagonals were measured at 30° to the left and right of the *Y*-axis in the measurement plane, with the longer diagonal set as LDA and the shorter diagonal set as SDB [2]. The calculation methods for the CI and CA were similar to those used for the caliper group.

#### 2.2.3. Inspection Accuracy Evaluation Using a Dummy Doll (Study 1)

The following procedures were performed using a Kyoto Kagaku (Kyoto, Japan) manufactured dummy newborn doll: one examiner recorded 10 measurements at appropriate time intervals (intra-examiner precision analysis), and then 10 examiners measured the same object (inter-examiner precision analysis). The mean and standard deviations for each measurement were calculated. The coefficient of variation (CV) was calculated (CV = standard deviation/mean).

#### 2.2.4. Measurement Accuracy in 1-Month-Old Infants (Study 2)

Two examiners performed two measurements each using the Mimos craniometer on healthy infants who visited the Kasukabe Medical Center for a 1-month checkup. The order of measurement was as follows: examiner 1 first time → examiner 2 first time → examiner 1 second time → examiner 2 second time. The same infants were analyzed using a stereophotogrammetry VECTRA H2 camera (Canfield Scientific, Parsippany-Troy Hills, NJ, USA). The infant’s head was protected using an elastic wig cap to prevent hair tangling, and then the head of the infant was photographed from nine directions (front, back, left, right, front-left, front-right, back-left, back-right, and top) using VECTRA H2 to create 3D-STL files.

The intra-class correlation coefficients (ICC) of the human-measured values were examined. Intra-examiner reliability was examined in ICC-Case 1 (ICC1,1). Furthermore, for inter-examiner reliability, the mean values of the measurements by examiners 1 and 2 were compared using ICC-Case 2 (ICC2,1).

**Figure 3:**
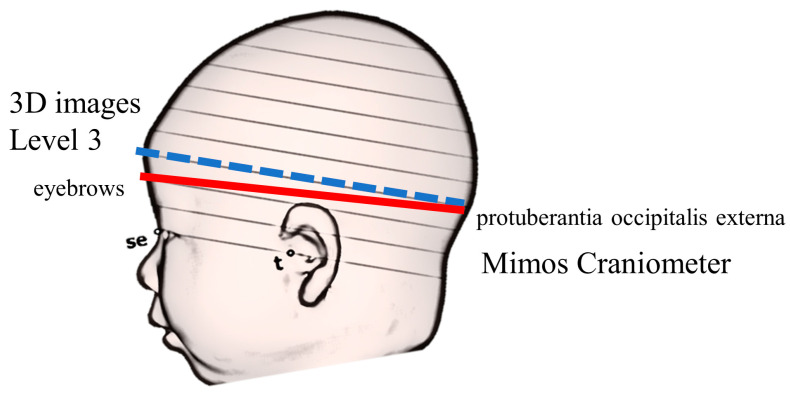
Difference between 3D images and caliper-measuring surfaces. This figure is a modified version of the figure prepared in our previous report [11]. The red line indicates the caliper-measuring surface, which is the circumference through the eyebrows and the protuberant occipitalis externa. The blue dotted line indicates the 3D image surface, which is the plane of level 3, horizontal to the reference plane. se, sellion; t, tragion.

Finally, we compared the mean human-measured values with 3D image measurements. Because the craniometer measurement plane was different from the measurement plane (level 3) in the 3D images (Figure 3) [11], the craniometer and 3DS measurements of the relative test, ICC-Case 3 (ICC3,1), were compared. Furthermore, the reproducibility of BC and DP diagnosis was examined using Cohen’s kappa coefficient (κ). The measurement plane of 3DS differs from that of the craniometer; this is the largest bias in this study. Several methods for determining the measurement plane of 3D images have been reported; however, at our facility, we chose level 3 [14,15,16]. Without the use of the 3DS, it is impossible to identify parallel level 3 planes in the actual cranium, because even if landmarks such as the sellion and the tragion are determined, there is no way to identify the planes that pass through these three points. Therefore, the measurement plane for the craniometer was set as the plane passing between the eyebrows and the protuberant occipitalis externa, which is used in conventional head circumference measurements. Therefore, the comparison between the 3DS measurements and craniometer measurements is only a relative comparison. Further, the goal of this study was not to diagnose DP but to screen for it.

#### 2.2.5. Database Comparison (Study 3)

We compared the symmetry-related parameters (CI and CA) established in a previous study using a database of healthy 1-month-old Japanese infants with those of affected 1-month-old infants measured using the Mimos craniometer [7,11]. To ensure similar characteristics in both groups, caliper matching was performed using markedly different values if there was a significant difference (*p* < 0.05) in characteristics. The caliper value was set at a standard deviation of 0.2.

The previously reported 3D parameters were obtained by scanning the head using the 3D scanner Artec Eva (Artec, Inc., Luxembourg, Luxembourg.) and converted into an STL file [7,10].

A two-tailed test was performed, and statistical significance was set at *p* < 0.05. Each parameter was subjected to Shapiro–Wilk normality test, where data with *p* < 0.05 were considered as having non-parametric data distribution. The Mann–Whitney U test was used to compare data with non-parametric distributions. Data with *p* ≥ 0.05 were classified as having normal data distribution. Student’s *t*-test was used to compare data with normal distributions. Furthermore, Fisher’s exact test was used to analyze the data in the form of ratios. Statistical analyses were performed using EZR software (version 1.61; Saitama, Japan) [17].

## 3. Results

During the study period, 129 infants at the age of one month visited any two hospitals to undergo measurements and examination during this study. After excluding four preterm infants and two non-Japanese infants, 123 infants were included in this study (study 3, caliper group). Both the VECTRA H2 and Mimos craniometer measurements were performed on 11 of the 123 participants (study 2).

### 3.1. Inspection Accuracy Evaluation Using Dummy Doll (Study 1)

The inspection accuracy results are listed in Table 1. The CVs of the actual measured values (CrL, CrW, LDA, and SDB) were <0.05, according to intra- and inter-examiner precision analyses.

### 3.2. Measurement Accuracy in 1-Month-Old Infants (Study 2)

During the study period, the cranium of 11 infants was measured using a Mimos craniometer and 3D scanner, with intra- and inter-examiner evaluations. The results are presented in Table 2.

Table 2A shows the intra-examiner comparisons and ICC (1,1). Intra-examiner reliability was found to be higher than 0.8 for all parameters. Table 2B shows the inter-examiner comparisons and ICC (2,1). Similarly, inter-examiner reliability values higher than 0.7 were observed for all parameters. Table 2C shows the comparison of the 3DS and craniometer (ICC 3,1) measurements, with inter-device reliability higher than 0.7, observed for all parameters except LDA and SDA. LDA and SDA did not achieve reliability. In addition, the reproducibility of agreement values between the BC and DP tests was κ = 1.0 and κ = 0.814, respectively (Table 3).

### 3.3. Comparison with the Database (Study 3)

The characteristics of 123 infants in the caliper group and 165 controls are shown in Table 4. The Shapiro–Wilk normality test showed that gestational age, birth weight, and measurement day were non-parametrically distributed. Maternal age and weight on measurement day were normally distributed. Age at measurement was used to perform caliper matching because of the significant differences between the groups (*p* < 0.01). The statistical software randomly selected 113 participants from each group, within the caliper range. After matching, there were no significant differences in age (median value: 32 days vs. 32 days; *p* = 0.99) and body weight (mean: 4166 g vs. 4139 g; *p* = 0.48) on the day measurements were performed. Additionally, no significant differences were observed in other parameters.

The symmetry-related parameters obtained after the matching are listed in Table 5. The Shapiro–Wilk normality test showed that all symmetry-related parameters were nonparametrically distributed. There were no significant differences in these parameters between the 3DS and caliper groups (CI, *p* = 0.98; CA, *p* = 0.48). In addition, there were no significant differences between the prevalence of BC and DP (BC: *p* = 0.35; DP: *p* = 0.89).

## 4. Discussion

The symmetry-related parameters of healthy 1-month-old infants were measured using a caliper and the 3DS results were compared. The caliper measurements of the CI and CA were not significantly different from those obtained using 3DS, and there were no accuracy concerns. Therefore, cranial shape measurements obtained using calipers are useful for screening BC and DP. This study is the first to compare 3DS measurements with those obtained using alternative methods in Japan.

### 4.1. Measurement Accuracy Using the Mimos Craniometer

The measurements from dummy doll and healthy 1-month-old infants were evaluated to provide similar levels of accuracy in this study. The Mimos craniometer is a simple instrument that uses an elastic band with a circumference of 310 mm wrapped around the head circumference cross section. Measurements were performed quickly between each symbol in the band. Measurements obtained using this method for CrL, CrW, LDA, and SDB had significant intra- and inter-examiner accuracies. In addition, Pastor-Pons et al. reported good intra- and inter-examiner accuracy for cranial shape determination using a Mimos craniometer in patients with DP [18]. The fact that we could follow up on these results, such as the high levels of accuracy in this examination of 1-month-old infants, confirms that the Mimos craniometer is a very useful and accurate tool.

### 4.2. 3D Image and Caliper Measurement Comparison

Traditionally, calipers, photographs, and drafting with landmarks have been used to determine cranial geometry without using 3DS [18,19,20,21]. However, with these methods, it can be difficult to accurately evaluate the left-to-right diagonal difference 30° from the *y*-axis, as proposed by Loveday et al. [2]. The position of the “+” symbol on the Mimos craniometer band indicated a partial circumference of 25 mm around a circle with a circumference of 310 mm, which (assuming a circle) would be at a central angle of approximately 29° (25 mm/310 mm × 360°). However, the actual shape of the cranium is not circular; it is oval-like, with CrL being the major diameter and CrW the minor diameter. Furthermore, as mentioned in the Methods section, because the 3DS and calipers measure different cross sections, the comparison of each parameter is relative. Therefore, the results for CrL and CrW, which have relatively small axis deviations, are reliable, but the results for LDA and SDB, which have large deviations and probably measure different axis lengths, are not reliable. However, the reliability of CA, the post-calculation parameters, was ensured (ICC (3.1): 0.738). These results suggest that DP can be evaluated by diagonal lines from the cranial center to the left and right sides at an equivalent position (whether at a central angle of 30° or at a partial circumference of 25 mm). Future studies should examine the consistency of the various evaluation methods for DP cases.

It was possible to determine craniometer measurements as relative rather than absolute values and use them for screening. Two studies comparing 3DS and caliper measurements of the same infants have been published, and both reported a strong correlation between 3DS and caliper measurements [22,23]. These results show that cranial geometry and screening for DP and BC are possible, even when using a caliper-based measurement method.

### 4.3. Necessity of Understanding the Cranial Shape at 1 Month of Age

A previous study examined the course of symmetry-related parameters in healthy infants and reported the worst CA values at three months and that the shape measured at one month subsequently occurred at six months. Moreover, a CA of 10 mm at one month was the threshold value for severe DP at six months [7]. Therefore, understanding the cranial shape at one month predicts the future cranial shape. Parents of infants with DP usually present to the hospital when the deformity is most visible; this usually occurs during the first three months of life. DP helmet therapy might be less effective if it is not started at six months of age [24,25]. It was considered that understanding the cranial shape at 1 month of age was useful in determining whether follow-up was possible at the first visit of a DP patient and whether helmet treatment should be started as early as 3 months of age, when cranial geometry is at its worst.

Previous studies have indicated that early interventions, such as positioning of the cranial geometry and the introduction of tummy time, could prevent DP [26,27]. In addition, with the introduction of the caliper method, we believe that the cranial shape during the first month of life could be ascertained, allowing early intervention. Although as many as 5% of healthy Japanese infants are potential candidates for helmet therapy, it is hoped that a simple measurement method that does not require 3DS will enable all infants in Japan to benefit from cranial shape assessments during health examinations [7].

### 4.4. Limitations

A limitation of this study is that it was only a screening test for DP and BC, and not suitable for the diagnosis of the clinical aspect of the skull (trapezoid vs. parallelogram shape, Harlequin orbit, etc.). The Mimos craniometer proved to be an excellent measurement device; however, it could not obtain parameters to assess the severity of DP, such as volume ratios, using a 3D scanner. It has been reported that 3D parameters, such as volume ratio, are more accurate than two-dimensional parameters, such as CA [28]. Therefore, future studies are required to compare the cranial volume ratio, a 3D parameter, with the parameters measured using the Mimos craniometer.

## 5. Conclusions

This study compared the accuracy of measurements obtained using a 3DS and a caliper, with similar levels of accuracy. The measurements obtained using calipers and elastic bands are useful for screening DP and BC. In the future, the widespread use of a simple screening method that can determine the craniofacial shape during examinations performed at the age of one month will enable early intervention and reduce the prevalence of DP and BC in the Japanese population.

## Figures and Tables

**Table 1:** Evaluation of inspection accuracy using a dummy doll.

		Intra-Examiner Analysis			Inter-Examiner Analysis		
		Mean	SD	CV	Mean	SD	CV
Cranial length	mm	107.3	0.64	0.006	107.0	1.11	0.01
Cranial width	mm	100.6	0.49	0.005	100.0	1.73	0.017
Longer diagonal A	mm	107.4	0.49	0.005	107.0	1.34	0.013
Shorter diagonal B	mm	106.7	0.46	0.004	105.9	1.37	0.013

CV, coefficient of variation; SD, standard deviation.

**Table 2:** Evaluation of inspection accuracy in 1-month-old infants.

**A**		Intra-Examiner Analysis (Craniometer) n:22				
		First Time	Second Time	ICC (1.1)	95% Confidence Interval	*p*-Value
Cranial length	mm	120.3 ± 4.1	120.3 ± 4.8	0.838	0.654–0.929	<0.01
Cranial width	mm	104.2 ± 4.4	104.2 ± 4.8	0.928	0.838–0.969	<0.01
Longer diagonal A	mm	118.6 ± 4.3	118.3 ± 4.4	0.964	0.916–0.985	<0.01
Shorter diagonal B	mm	113.3 ± 3.5	113.6 ± 3.6	0.904	0.786–0.959	<0.01
Cephalic index	%	86.7 ± 4.9	86.8 ± 5.7	0.862	0.700–0.940	<0.01
Cranial asymmetry	mm	5.7 ± 4.0	5.2 ± 3.6	0.898	0.773–0.956	<0.01
**B**		Inter-examiner analysis (Craniometer) n:11				
		Examiner 1	Examiner 2	ICC (2.1)	95% Confidence Interval	*p*-value
Cranial length	mm	120.3 ± 4.0	120.3 ± 4.7	0.845	0.516–0.956	<0.01
Cranial width	mm	104.4 ± 4.4	104.0 ± 4.8	0.927	0.759–0.960	<0.01
Longer diagonal A	mm	118.4 ± 4.4	118.5 ± 4.5	0.870	0.584–0.963	<0.01
Shorter diagonal B	mm	113.8 ± 3.3	113.4 ± 3.7	0.701	0.203–0.910	<0.01
Cephalic index	%	86.9 ± 5.1	86.6 ± 5.4	0.938	0.791–0.983	<0.01
Cranial asymmetry	mm	4.6 ± 3.4	5.1 ± 3.9	0.860	0.577–0.996	<0.01
**C**		Inter-device analysis (Craniometer vs. VECTRA H2) n:11				
		Craniometer	VECTRA H2	ICC (3.1)	95% Confidence Interval	*p*-value
Cranial length	mm	120.3 ± 4.2	125.9 ± 4.9	0.718	0.243–0.915	<0.01
Cranial width	mm	104.2 ± 4.5	109.3 ± 4.8	0.939	0.789–0.983	<0.01
Longer diagonal A	mm	118.4 ± 4.3	118.8 ± 10	0.253	−0.378–0.723	0.21
Shorter diagonal B	mm	113.6 ± 3.2	114.6 ± 8.7	0.086	−0.516–0.631	0.40
Cephalic index	%	86.7 ± 5.2	86.9 ± 5.2	0.931	0.764–0.981	<0.01
Cranial asymmetry	mm	4.8 ± 3.5	4.1 ± 3.4	0.738	0.283–0.922	<0.01

Data are presented as mean ± standard deviation and number (%). ICC: intraclass correlation coefficients.

**Table 3:** Evaluation of inspection accuracy in Cohen’s kappa coefficient.

		Craniometer	VECTRA H2	Cohen’s Kappa Coefficient	95% Confidence Interval
Number		11	11		
Brachycephaly	n (%)	4 (36.4)	4 (36.4)	1.000	1.000–1.000
Deformational plagiocephaly	n (%)	5 (45.5)	4 (36.4)	0.814	0.465–1.162

Data are presented as number (%). 3DS: three-dimensional scanner.

**Table 4:** Comparison of participant characteristics.

		Control (3DS)	Caliper	*p*-Value	
Participants, n		165	123		
Age of the mother	years	33.1 ± 5.2	33.2 ± 5.3	0.94	*
First child	n (%)	80 (48.5)	53 (43.1)	0.40	***
Gestational age	weeks	39.0 (37.9–40.0)	38.9 (37.8–39.9)	0.44	**
Birth weight	g	3008 (2820–3260)	3005 (2726–3238)	0.34	**
Male	n (%)	89 (53.9)	61 (49.6)	0.48	***
Measurement day age	days	34 (31–40)	31 (30–34)	<0.01	**
Weight on measurement day	g	4291 ± 529	4133 ± 508	0.01	*

*: normal distribution; data are presented as mean ± standard deviation; Student *t*-test. **: non-parametric distribution; data are presented as median (interquartile range); Mann–Whitney U test. ***: number (%); Fisher’s exact test. 3DS: three-dimensional scanner.

**Table 5:** Comparison of symmetry-related parameters after matching.

		Control (3DS)	Caliper	*p*-Value	
n		113	113		
Measurement day age	days	32 (30–34)	32 (30–34)	0.98	**
Weight on measurement day	g	4166 ± 739	4139 ± 509	0.48	*
Cephalic index	%	85.0 (80.8–89.0)	85.2 (80.6–89.1)	0.98	**
Cranial asymmetry	mm	5.9 (3.5–8.4)	6.0 (3.0–9.0)	0.48	**
Brachycephaly	n (%)	14 (12.4)	20 (17.7)	0.35	***
Deformational plagiocephaly	n (%)	66 (58.4)	64 (56.6)	0.89	***

*: normal distribution; data are presented as mean ± standard deviation; Student *t*-test. **: non-parametric distribution; data are presented as median (interquartile range); Mann–Whitney U test. ***: number (%); Fisher’s exact test. 3DS: three-dimensional scanner.

## Data Availability

The data presented in this study are available on request from the corresponding author.
